# Best implant choice for coracoid graft fixation during the Latarjet procedure depends on patients’ morphometric considerations

**DOI:** 10.1186/s40634-020-00230-0

**Published:** 2020-03-17

**Authors:** Achilleas Boutsiadis, Ioannis Bampis, John Swan, Johannes Barth

**Affiliations:** 1 Department of Orthopedic Surgery, 401 Military Hospital of Athens, Athens, Greece; 2 Department of Orthopaedic Surgery, Centre Osteoarticulaire des Cèdres, Parc sud galaxie, 5 Rue Des Tropiques, 38130 Echirolles, Grenoble, France

**Keywords:** Coracoid dimensions, Glenoid dimensions, Latarjet technique, Latarjet implants, Glenoid bone loss

## Abstract

**Purpose:**

To assess the anthropometric dimensions of the coracoid process and the glenoid articular surface and to determine possible implications with the different commercially available Latarjet fixation techniques.

**Methods:**

In a total of 101 skeletal scapulae the glenoid length (GL), the glenoid width (GW), the coracoid length (CL), the coracoid width (CW) and the coracoid thickness (CTh) were measured. In order to assess the ability of the transferred coracoid to restore the glenoid anatomy we created a hypothetical model of 10%, 15%, 20%, 25% and 30% glenoid bone loss. We analyzed four common surgical fixation techniques for the Latarjet procedure (4.5 mm screws, 3.75 mm screws, 3.5 mm screws, and 2.8 mm button). The distances from the superior-inferior and medio-lateral limits of the coracoid using the four different fixation methods were calculated. We hypothesized that the *“safe distance”* between the implant and the coracoid osteotomy should be at least equal to the diameter of the implant.

**Results:**

The intra and inter-observer reliability tests were almost perfect for all measurements. The mean GH was 36.8 ± 2.5 mm, the GW 26.4 ± 2.2 mm, the CL 23.9 ± 3 mm, the CW 13.6 ± 2.mm, and the mean CTh was 8.7 ± 1.3 mm. The CL was < 25 mm in 46% of the cases. In cases with 25% and 30% bone loss, the coracoid graft restored the glenoid anatomy in 96% and 79.2% of the cases. With the use of the 4.5 mm screws the *“safe distance”* was present in 56% of the cases, with the 3.75 mm screws in 85%, with the 3.5 mm screws in 87%, and with the 2.8 mm button in 98% of the cases. The distance from the medio-lateral limit of the coracoid could be significantly increased (up to 9 mm) when smaller-button implants are used.

**Conclusions:**

The coracoid graft could not always restore glenoid defects of 30%. Larger implants could be positioned too close to the osteotomy and the “medio-lateral offset” of the coracoid could be increased with smaller implants.

## Introduction

The glenohumeral joint is the least stable joint in the human body, and is prone to recurrent anterior instability. Anterior shoulder instability is disabling and has the highest incidence in young males, and the preferred treatment is surgical [1]. The two commonest surgical treatments for anterior shoulder instability are the arthroscopic Bankart repair and the Latarjet procedure [2].

The Latarjet procedure was first described in 1954 [3], and the advantage of this technique is the triple blocking stabilizing mechanism proposed by Patte and Debeyre: a) the “bone block effect”, b) the “ligament effect” of the repair of the capsule to the stump of the coracoacromial ligament, and c) the “sling effect” that is produced by the dynamic interaction between the subscapularis muscle and the conjoint tendon [4]. Biomechanical studies have shown that the dynamic “sling effect” may be the most important stabilizing factor [5]. Recent studies have reported that the Latarjet procedure may be indicated even in cases with minimal glenoid bone loss, and could have good to excellent long term results [2, 6–8]. However, several reports highlighted the completely different treatment approach of the French surgeons, who in 72% of cases prefer the Latarjet procedure as the primary treatment option, while other international surgeons preferred the Bankart repair in 90% of primary cases [9]. A possible explanation for this observation may be the significant technical difficulties and the possible complications of the original Latarjet technique [10, 11]. Some of the complications, such as coracoid graft fracture or postoperative recurrence could be related to specific osseous anatomy of the glenoid and coracoid process [10]. For example, when performing the open Latarjet procedure recommended by Walch, a coracoid bone graft length of more than 25 mm is necessary to enable the safe insertion of two 4.5 mm screws [11] or when the glenoid erosion is excessive, a “congruent arc” technique is necessary [12].

## Purpose

The purpose of this study was, firstly to assess the anthropometric dimensions of the coracoid process and the glenoid articular surface in the young Greek population and secondly, to determine possible implications with the different commercially available Latarjet fixation methods.

## Methods

This study did not include live human subjects and therefore did not require Investigational Review Board or Ethical Committee approval.

A total of 163 intact skeletons of Greek male soldiers, who died during the Second World War, were examined by an anthropologist. Study inclusion criteria were scapulae with intact coracoid and glenoid anatomy and estimated age at the time of death between 20 and 30 years old with corresponding information, including gender and ethnicity. Exclusion criteria were the presence of any bone loss or osteophytes that could affect measurement reliability. Α total of 101 skeletally mature bone specimens of scapulae from 97 skeletons were included in the study (4 skeletons had bilateral scapulae included).

**Fig. 1:**
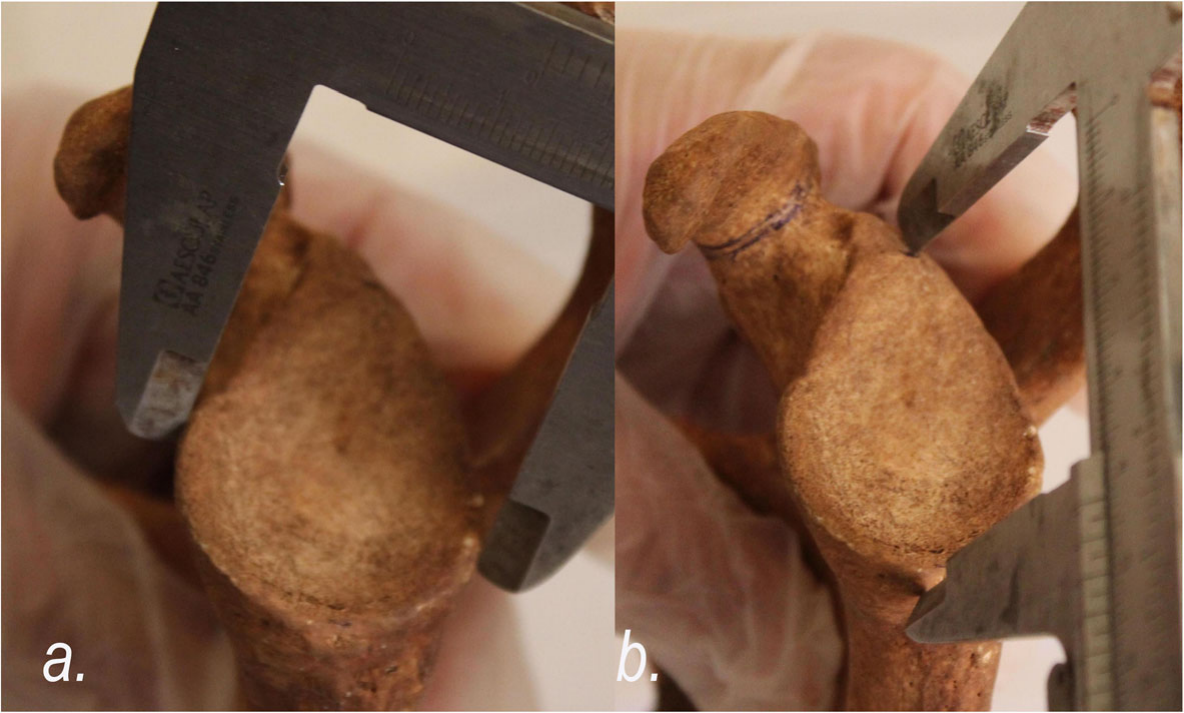
The glenoid maximum (**a**) anterior-posterior (glenoid width - GW) and (**b**) the superior-inferior dimension (glenoid height - GH) are measured

**Fig. 2:**
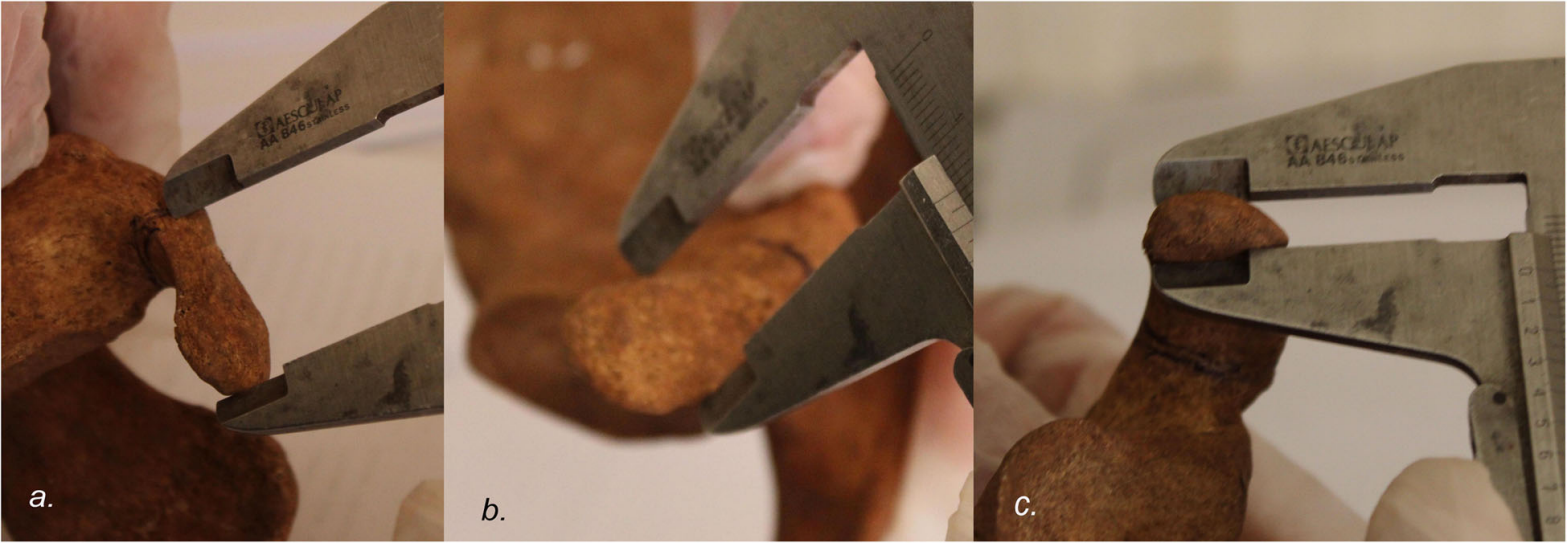
The coracoid (**a**) anterior-posterior (coracoid length - CL), (**b**) medial-lateral (coracoid width - CW) and (**c**) superior-inferior (coracoid thickness - CTh) are measured

The glenoid anterior-posterior dimension (glenoid width - GW) was measured at the point of greatest width, and the superior-inferior dimension (glenoid height - GH) was measured from the supraglenoid to the infraglenoid tubercle (Fig. 1). The coracoid was measured in three perpendicular dimensions: anterior-posterior (coracoid length - CL), medial-lateral (coracoid width - CW) and superior-inferior (coracoid thickness - CTh) (Fig. 2). The coracoid length was measured from the tip to where the inferior cortex curves inferiorly (knee of the coracoid). The coracoid thickness and width were measured at a point 10 mm from the coracoid tip [13]. At this point was also located the thinnest part the coracoid. Therefore, we selected this point in order to mimic the coracoid thickness after flattening its inferior surface intra-operatively. All the measurements were made using a hand-held Vernier analog caliper with nominal precision of 0.1 mm. In order to assess the intra-and inter-observer reproducibility of our measurements, 20 specimens were initially randomly chosen and measured by two of the authors.

**Fig. 3:**
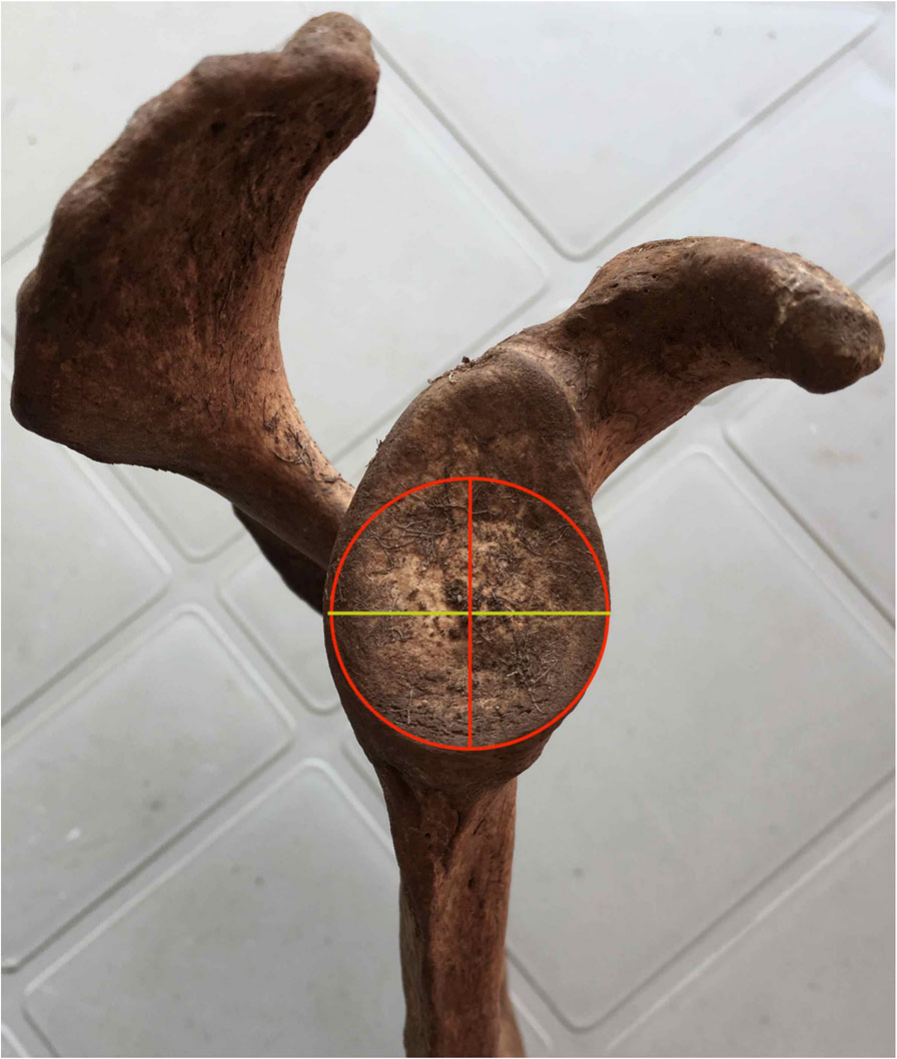
The hypothetical glenoid bone loss was calculated using the Diameter-Based Method as follows: Percent bone loss = (Defect width/Diameter of inferior glenoid circle) × 100%

In order to assess the ability of the transferred coracoid to restore the width of the glenoid during the classic Latarjet technique we created a hypothetical bone model and we calculated the glenoid width in the setting of 10%, 15%, 20%, 25% and 30% bone loss (Fig. 3). We next calculated the ratio of the bone available to restore the glenoid loss by dividing the coracoid thickness by the amount of bone lost in 10%, 15%, 20%, 25% and 30% bone loss scenarios.

Furthermore, we chose to analyze four common surgical fixation techniques for the Latarjet procedure:

- the Arthrex 3.75 mm titanium cannulated screw (Arthrex, Naples, FL, USA)
- the Mitek 3.5 mm titanium cannulated Bristow–Latarjet Instability Shoulder Screw (Depuy Synthes Mitek Sports Medicine, Raynham, MA, USA)
- the Synthes 4.5 mm steel Large Fragment LCP System Malleolar Screw (Synthes, West Chester, PA, USA)
- the 2.8 mm Smith & Nephew Round Button Latarjet Technique (Smith + Nephew, Inc. Andover, USA).

**Fig. 4:**
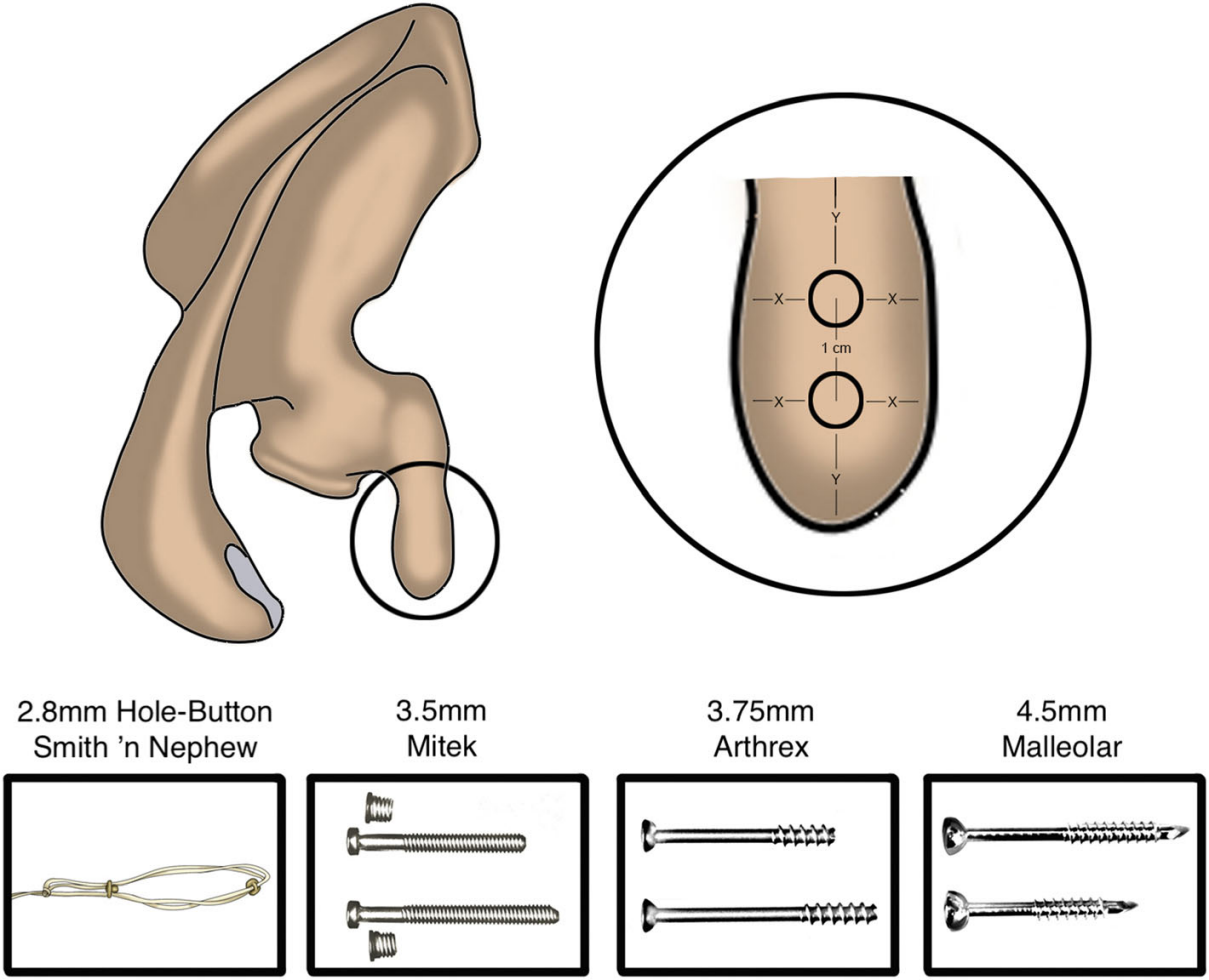
Art design showing the harvested coracoid graft. The distance from the center of the two holes was set at 10 mm. Y = Distance from the Supero-Inferior Limit. X = Distance from the Medio-Lateral Limit. Also are shown the four common surgical fixation techniques for the Latarjet procedure

In our hypothetical model, two implants (screws or buttons) were used to fix the coracoid to the glenoid, and the distance between the two holes was set at 10 mm for all types of fixation (Fig. 4). Thereafter, the distances from the superior-inferior and medio-lateral limits of the coracoid during the Latarjet procedure were calculated as follows:

1. Distance from the Supero-Inferior Limit = [CL-(10 + Implant Diameter)]/2
2. Distance from the Medio-Lateral Limit = (CW-Implant Diameter)/2.

Furthermore, according to the AO principles, at the apex of a fracture fragment, the minimal distance between the screw head and the fracture line must be at least equal to the diameter of the screw head [14]. In the setting of the Latarjet procedure we hypothesized that the *“safe distance”* between the implant used and the coracoid osteotomy point should be at least equal to the diameter of the implant.

### Statistical analysis

Continuous data were reported as mean with standard deviation and range. Categorical data were reported as percentages. Based on the Shapiro-Wilk test, major deviations from normality were revealed. Thereafter, continuous variables were compared with the nonparametric Spearman correlation or the Mann-Whitney U test, and categorical variables were compared with the chi-square test.

Intra and inter-observer agreement for GW, GH, CL, CW and CTh were evaluated by the intraclass correlation coefficient (ICC or Kendall coefficient) 2-by-2 with a 95% confidence interval. The power of ICC values was interpreted according to the Landis and Koch classification as no agreement to slight agreement, < 0.20; fair agreement, 0.21 to 0.40; moderate agreement, 0.41 to 0.60; substantial agreement, 0.61 to 0.80; and almost perfect agreement, 0.81 to 1.00.

Statistical significance was set at *p* < .05, and the analysis was performed using SPSS (v 25.0; IBM Corp).

## Results

### Intra and inter-observer reliability tests

The intra and inter-observer agreement for the GH and GW were almost perfect. The intra and inter-observer agreement for the CL, CW and CTh were substantial to almost perfect (Table 1).

**Table 1:** Intra- and inter-observers agreements measured by the Intraclass Correlation Coefficient (ICC), 2 by 2 with 95% of confidence interval

	Intra-observer agreement	Inter-observer agreement
	ICC value (95% CI)	ICC value (95% CI)
**Glenoid Height (GH)**	0.9 (0.85–0.94)	0.88 (0.73–0.94)
**Glenoid Width (GW)**	0.91 (0.87–0.93)	0.89 (0.84–0.93)
**Coracoid Length (CL)**	0.85 (0.74–0.89)	0.82 (0.74–0.87)
**Coracoid Width (CW)**	0.91 (0.85–0.95)	0.88 (0.84–0.93)
**Coracoid Thickness (CTh)**	0.89 (0.79–0.93)	0.91 (0.86–0.94)

### Glenoid and coracoid dimensions

The mean GH was 36.7 ± 2.5 mm (range 43-30 mm) and the mean GW was 26.4 ± 2.2 mm (range 22-33 mm). The mean CL was 23.9 ± 3 mm (range 14-30 mm), the mean CW was 13.6 ± 2 mm (range 6-21 mm), and the mean CTh was 8.7 ± 1 mm (range 5.5-13 mm). The CL was in 46% of the cases < 25 mm (21.4 ± 2 mm, range 14–23.5 mm).

**Fig. 5:**
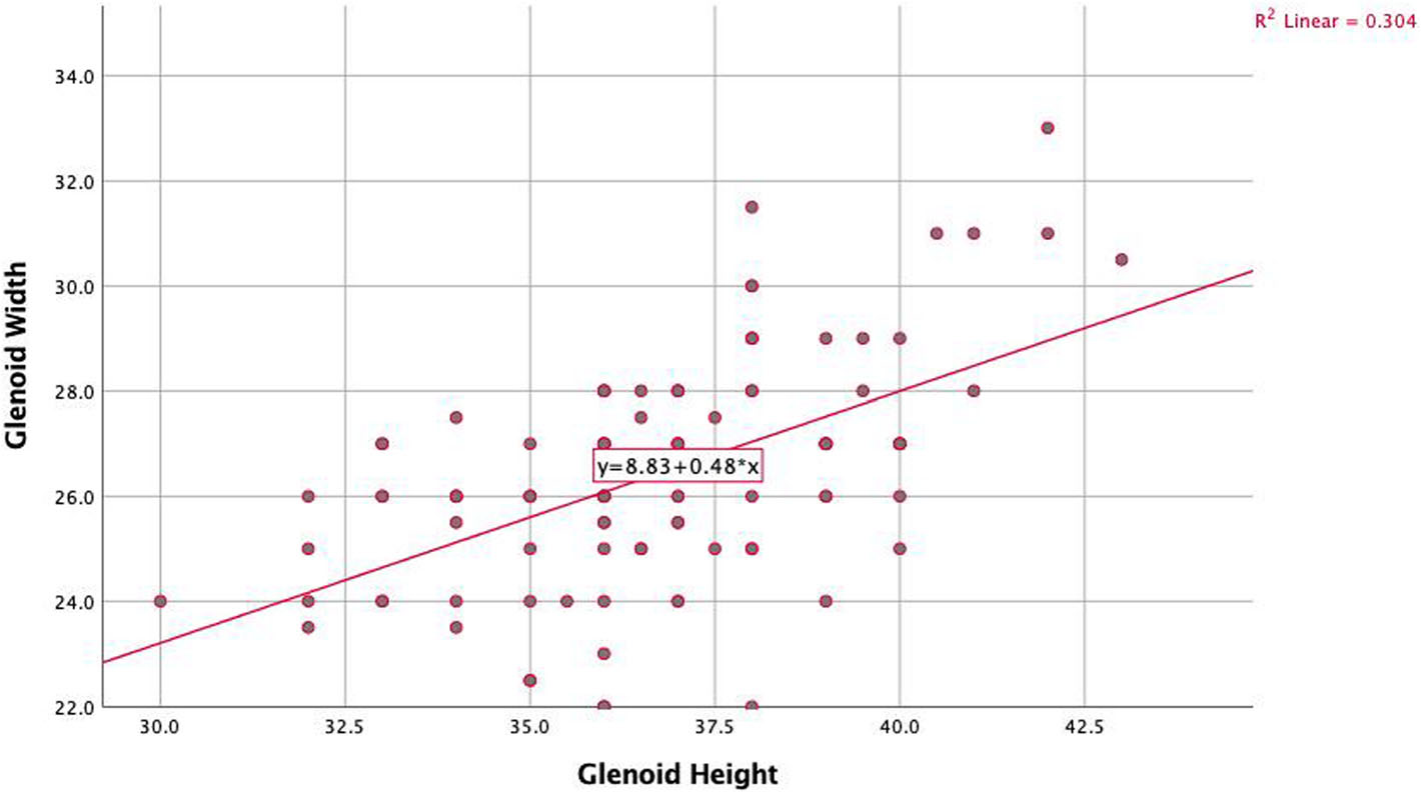
A strong positive correlation between the height and the width of the glenoid was observed (rho = 0.53, *P* < .001)

A strong positive correlation between the height and the width of the glenoid was observed (rho = 0.53, *P* < .001) (Fig. 5). Regarding the coracoid, a positive correlation was found between its length (CL) and width (CW) (rho = 0.24, *p* = 0.016). No correlations were found between the CTh and the CL (rho = 0.145, *p* = 0.15) and the CTh and the CW (rho = 0.19, *p* = 0.06).

**Fig. 6:**
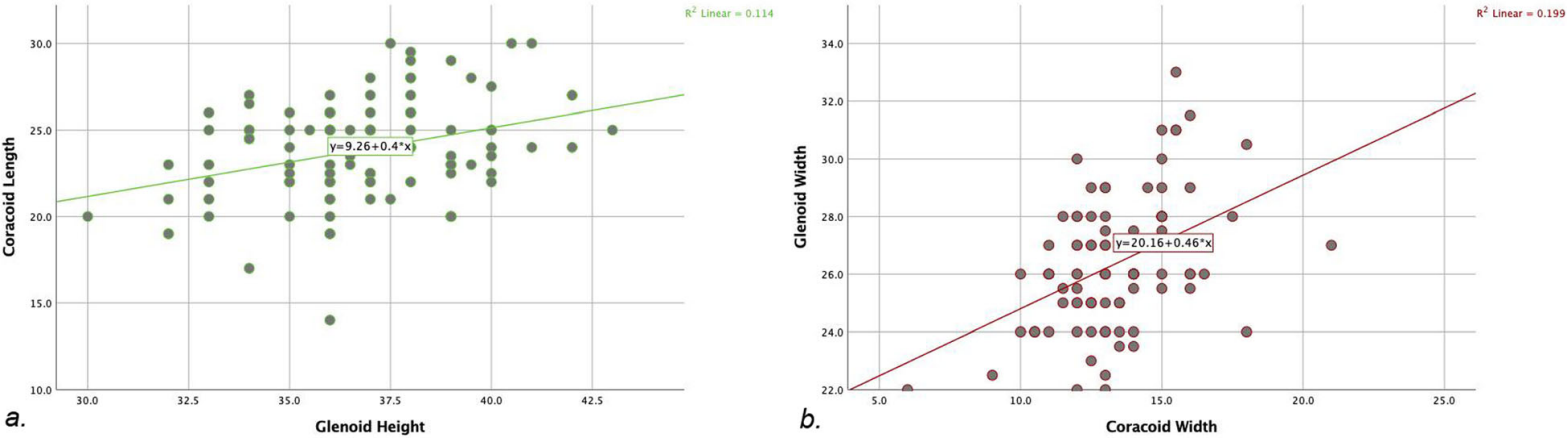
A positive correlation was found between (**a**) the GH and the CL (rho = 0.3, *p* = 0.002) and (**b**) between the GW and the CW (rho = 0.45, *p* < 0.001)

Furthermore, a positive correlation was found between the GH and the CL (rho = 0.3, *p* = 0.002) and between the GW and the CW (rho = 0.45, *p* < 0.001) (Fig. 6a, b). No correlations were observed between the GW and the CTh (rho = 0.135, *p* = 0.181).

### Glenoid bone loss restoration

In all cases with 10%, 15% and 20% of glenoid bone loss, the coracoid graft positioned with the classic Latarjet technique was able to restore glenoid width. The respective ratios are shown in Table 2. Also, in cases with 25% and 30% bone loss, the coracoid graft was thick enough to restore the glenoid anatomy in 96% and 80% of the cases. The ratios are also shown in Table 2.

**Table 2:** Overall results according to Glenoid Bone Loss

***Glenoid Bone Loss***	***10%***	***15%***	***20%***	***25%***	***30%***
Restoration of Glenoid Anatomy (Percentage of Cases)	100%	100%	100%	96%	76.2%
Bone Coverage Ratios	3.3 ± 0.6 (2.3–5.4)	2.2 ± 0.4 (1.5–3.6)	1.7 ± 0.3 (1.2–2.7)	1.3 ± 0.2 (0.9–2.2)	1 ± 0.03 (0.8–1.8)

### Implications for different Latarjet techniques

The distance from the superior or inferior border of the coracoid of the 4 different implants types, according to the technique used, are presented in Table 3. With the use of the 2.8 mm Smith + Nephew Round Button the “safe distance” was ensured in 98% of the cases. With the use of the originally proposed 4.5 mm malleolar screws this “safe distance” was present in 56% of the cases, with the 3.75 mm Arthrex screws in 85% and with the Mitek 3.5 mm screws in 87% of the cases respectively.

**Table 3:** Distance from Coracoid Borders According to Implant Type

	2.8 mm Smith ‘n Nephew	3.5 mm Mitek Depuy	3.75 mm Arthrex	4.5 mm Malleolar	*P* values
Supero-Inferior Distance	5.5 ± 1.5 mm (0.6 to 8.6 mm)	5.2 ± 1.5 mm (0.3 to 8.3 mm)	5 ± 1.5 mm (0.1 to 8 mm)	4.7 ± 1.5 mm (−0.3 to 7.8 mm)	*P* < 0.001^#^
Medio-Lateral Distance	5.4 ± 1 mm (1.6 to 9.1 mm)	5 ± 1 mm (1.3 to 8.8 mm)	4.9 ± 1 mm (1.1 to 8.6 mm)	4.5 ± 1 mm (0.8 to 8.3 mm)	*P* < 0.001^#^

# = *p* value of Friedman test

Furthermore, the distance of the 4 different implants from the medio-lateral borders of the coracoid are also presented in Table 3.

## Discussion

The first main finding of our study was that the mean coracoid bone graft length available for transfer (from the coracoid tip to where the inferior cortex curves inferiorly) in our young population was 23.9 ± 3 mm. Therefore, in 46% of the cases there was less than the ideal length of 25 mm, according to the original technique [11, 15]. Previous reports have shown divergent results regarding the maximal harvestable coracoid length. Dolan et al. and Shibata et al. reported in cadaver studies that the mean maximum length available for transfer was 28.5 mm [16] and 27 mm [17] respectively. Furthermore, Paladini et al. in computed tomography study and Young et al. in an intra-operative measurement study, found the available coracoid length to be 26.3 mm and 26.4 mm respectively [18]. However, Bhatia et al. and Lian et al. reported values of 19 mm and 24 mm respectively [19, 20]. From the aforementioned studies and the international literature, the coracoid length seems to be positively affected by male gender, age, overall height and Caucasian genetics [12, 13, 17, 20, 21]. In our study population, the height of the cadavers was not available, however we observed a positive correlation with the glenoid height.

The second main finding was that the harvestable coracoid length directly affects the position and possibly the biomechanics of the different implants used during fixation in the Latarjet procedure. Based on the AO principles we hypothesized that when the distance between the implant and the coracoid osteotomy is at least equal to the diameter of the implant, it would be “safe”, with low fracture risk. Therefore, complications such as acute coracoid fracture or later osteolysis could be avoided [10]. With the use of the originally proposed 4.5 mm malleolar screws this “safe distance” was ensured only in 56% of the cases. This percentage was significantly improved with smaller screws or the use of cortical button fixation. Despite this, no studies exist comparing the different types of fixation. This could explain the short-term differences in coracoid fracture and osteolysis when 4.5 mm screw fixation [22, 23] is compared to button fixation [24, 25]. However, when Boileau et al. perform their Latarjet technique, only 15 mm of the coracoid is harvested and fixation is with only one button [24]. In our study, for homogeneity reasons, we created a hypothetical model in which the complete coracoid is harvested and there are two points of fixation.

Furthermore, we found that the coracoid graft thickness is able to restore the glenoid anatomy in most of the cases when the classic Latarjet technique was performed. The mean “filling ratio” could be 3.3 to 1.7 in cases with smaller glenoid bone loss (10% to 20%) and 1.3 in most of the cases with defects of 25%. This extension of concavity of the glenoid articular arc may better manage the bipolar-“off-track” lesions [26] and explains the favorable clinical outcomes and the lower recurrence rates when the Latarjet procedure is performed in cases with even 13.5% glenoid bone loss [6, 27]. However, we found that in cases with 30% glenoid bone loss, the coracoid graft was not always enough to achieve a “filling ratio” of 1.0 (20% of the cases). Hantes et al. reported similar results in their cadaveric study. In intact glenoids with a mean area of 734 ± 89 mm^2^, they created a defect of 29%. After the reconstruction, the mean surface area of the glenoid was still smaller (708 ± 71 mm^2^), but this was not statistically significant [28]. Furthermore, the authors found that the coracoid thickness represents 27 ± 5% of the intact glenoid [28].

In a clinical study, Moon et al. operated on 44 patients with large glenoid defects of 25.3% ± 6% of the intact glenoid surface. Using 3-D CT-scans, they found 1.5 ± 2% recurrent bone defect, however, this did not affect the clinical outcome [29]. Also, Paladini et al., in 23 patients with glenoid defects greater than 20%, found that the coracoid filled the defect by 102% [18]. These small differences in filling the defect in published studies could be due to differences in age, race and patient height [13, 20]. However, when the glenoid bone loss is ≥30% the “congruent arc”- modified Latarjet [12] or the Eden-Hybinette procedure [7] could be preferred. Regarding the “congruent arc” technique, our results also showed that it may be indicated in greater defects, as the coracoid width was always greater that the coracoid thickness.

Young et al. performed the classic Latarjet technique with 4.5 mm malleolar screws, and during their intra-operative measurements, found that the distance from the edge of the inferior drill hole to the lateral margin of the graft was 5.7 ± 1.1 mm [30]. Sahu et al., also using 4.5 mm screws in their cadaveric study, found that this “lateral offset” of the coracoid graft was 5.5 ± 1 mm [31]. Our study results were similar, when using screw fixation. However, surgeons should be careful when the drill holes are placed in the middle of the coracoid and smaller implants are used. In these cases the “lateral offset” could be increased (by up to 9 mm).

### Strengths and limitations

In this anthropometric study we tried to describe the possible implications between the coracoid process dimensions and the different surgical techniques. A strength of this study is the young male population (soldiers from the Second World War) that corresponds to the type of patients commonly operated on for shoulder instability. However, we cannot take into account the evolution of the Greek population over time, and nowadays larger glenoid and coracoid dimensions could be present within the population. Despite the measurements being reproducible (intra and inter-observer reliability), this was not a cadaveric study and the soft tissue insertions (pectoralis minor, coracoclavicular and acromioclavicular ligaments) were not present. Also, other anthropometric details of the specimens (like the height) were not available. The glenoid bone loss and the application of different materials were performed by using a “hypothetical” model and not in real practice. Finally, for homogeneity reasons all measurements were performed regarding the classic Latarjet technique and not the congruent arc or the Bristow technique.

## Conclusions

The harvested coracoid graft during the classic Latarjet procedure is adequate to restore up to 25% glenoid defects. However, in cases with bone loss greater than 30%, the coracoid graft is not always enough to reconstruct the normal surface area of the glenoid. Furthermore, in 46% of our specimens the harvestable coracoid length was smaller that 25 mm. Therefore, surgeons should be cautious when using larger implants (4.5 mm screws) that could be positioned too close to the osteotomy, creating a fracture risk. Finally, the distance from the medio-lateral limit of the coracoid could be significantly increased (up to 9 mm) when smaller-button implants are used. The Latarjet procedure is technically demanding and the pre and peri-operative planning with careful measurement of the glenoid and coracoid dimensions is mandatory for clinical success.

## Abbreviations

GL: Glenoid Length
GW: Glenoid Width
CL: Coracoid Length
CW: Coracoid Width
CTh: Coracoid Thickness
